# Trends and disparities in neoplasm-related mortality with death-certificate-documented protein-energy malnutrition in the United States, 1999–2024: a CDC WONDER multiple cause of death study

**DOI:** 10.3389/fnut.2026.1860679

**Published:** 2026-07-28

**Authors:** Qi Gan, Kaide Xia, Xiuju Yang

**Affiliations:** 1 Department of Anesthesiology, Guizhou Aerospace Hospital, Zunyi, China; 2 Central Laboratory, Guiyang Maternal and Child Health Care Hospital, Guiyang Children's Hospital, Guiyang, China

**Keywords:** CDC WONDER, health disparities, mortality trends, multiple cause of death, neoplasms, protein-energy malnutrition

## Abstract

**Background:**

Malnutrition is clinically important but often underrecognized among patients with neoplasms. National temporal patterns of neoplasm-related deaths with death-certificate-documented protein-energy malnutrition (PEM) remain unclear.

**Methods:**

We conducted a population-based serial cross-sectional study using CDC WONDER Multiple Cause of Death data. Deaths among persons aged ≥15 years with neoplasms as the underlying cause (ICD-10 C00–D48) and PEM in multiple-cause-of-death fields (E40–E46) were identified for 1999–2024. Annual death counts and age-adjusted mortality rates (AAMRs) per 100,000 population, standardized to the 2000 US population, were assessed using Joinpoint regression. Trends were examined overall and across demographic, geographic, neoplasm-category, and state strata. We also calculated the annual proportion of all neoplasm-related deaths with documented PEM.

**Results:**

Annual neoplasm-related deaths with death-certificate-documented PEM increased from 5,024 in 1999 to 18,410 in 2024, while the AAMR increased from 2.32 (95% CI, 2.26–2.38) to 5.26 (95% CI, 5.19–5.34) per 100,000 population. The proportion of all neoplasm-related deaths with documented PEM increased from 0.90% (95% CI, 0.87%−0.92%) to 2.90% (95% CI, 2.86%−2.94%). Joinpoint analysis identified inflection points in 2006 and 2013, with an early decline, an intermediate stable period, and a marked subsequent increase; the overall AAPC was 3.54%. In 2024, AAMRs were higher among males than females (6.32 vs. 4.43), Black than White individuals (6.42 vs. 5.32), and adults aged ≥65 years than those aged 15–64 years (24.47 vs. 1.58 per 100,000). Nonmetropolitan areas had higher rates than metropolitan areas through 2020. Digestive neoplasms represented the largest observed broad-category burden in both years.

**Conclusion:**

Death-certificate-documented PEM among neoplasm-related deaths increased substantially in the United States from 1999 to 2024 and showed marked demographic, geographic, and neoplasm-category disparities. The rising proportion of all neoplasm-related deaths with documented PEM suggests that the trend was not explained solely by changes in overall neoplasm mortality. However, these descriptive data cannot distinguish changes in underlying nutritional vulnerability from changes in clinical recognition and death-certificate documentation.

## 1 Introduction

Malnutrition is a common but frequently underrecognized condition among patients with neoplasms, particularly those with advanced disease, a high inflammatory burden, impaired oral intake, gastrointestinal dysfunction, or cancer-associated cachexia (1–5). Nutritional deterioration in this population is an important prognostic factor and has been consistently associated with poorer treatment tolerance, increased treatment-limiting toxicities, functional decline, reduced quality of life, and a higher risk of mortality (6–10). Accordingly, proactive nutritional assessment and support are increasingly regarded as essential components of comprehensive oncologic and supportive care (11–15).

Despite its clinical importance, the population-level mortality burden of nutritional compromise among individuals dying from neoplasms remains insufficiently characterized (6, 8, 9). Although previous studies based on single-center cohorts, disease-specific clinical samples, or selected hospitalized populations have provided important clinical insights, they do not fully capture long-term national trends, demographic disparities, geographic variation, or heterogeneity across major neoplasm categories (8, 16, 17). In particular, little is known about how the mortality burden associated with death-certificate-documented protein-energy malnutrition has evolved over time at the national level in the United States (3, 17).

Death certificate data provide a complementary population-level perspective on this issue (18, 19). In the present study, protein-energy malnutrition recorded on death certificates likely underestimates the true prevalence of nutritional compromise, including milder malnutrition, cachexia, sarcopenia, and unintentional weight loss, but may serve as a relatively specific indicator of severe, clinically recognized nutritional vulnerability near the end of life (20, 21). Accordingly, multiple-cause-of-death data offer a useful framework for examining the long-term burden and temporal trends of neoplasm-related deaths with death-certificate-documented protein-energy malnutrition, as well as disparities across sex, race, age, region, urbanization, and state (18, 19).

Therefore, using the Centers for Disease Control and Prevention Wide-ranging ONline Data for Epidemiologic Research (CDC WONDER) Multiple Cause of Death database, we examined neoplasm-related deaths with death-certificate-documented protein-energy malnutrition in the United States from 1999 to 2024. The objectives of this study were to: quantify the overall burden and temporal trends in age-adjusted mortality rates (AAMRs); assess demographic and geographic disparities; evaluate heterogeneity across major neoplasm categories; and describe state-level variation in both contemporary mortality burden and long-term rate changes.

## 2 Methods

### 2.1 Data source and study design

We conducted a population-based, serial cross-sectional study using the CDC WONDER Multiple Cause of Death database. To ensure complete temporal coverage, mortality data for 1999–2020 were obtained from the “Multiple Cause of Death, 1999–2020” bridged-race dataset, and data for 2021–2024 were obtained from the “Multiple Cause of Death, 2018–2024, Single Race” dataset (22). Because these data systems differ in race classification and population denominators, we performed temporal robustness analyses restricted to the bridged-race period and compared bridged-race and single-race estimates during their 2018–2020 overlap period. All data were publicly available and deidentified; therefore, institutional review board approval and informed consent were not required. This study was reported in accordance with the Strengthening the Reporting of Observational Studies in Epidemiology (STROBE) reporting guideline.

### 2.2 Study population and variables

We identified deaths among individuals aged 15 years and older for whom neoplasms were listed as the underlying cause of death (UCD), defined using ICD-10 codes C00–D48 (23). Death-certificate-documented protein-energy malnutrition was defined as the presence of ICD-10 codes E40–E46 in any of the multiple-cause-of-death fields (24). Accordingly, the analytic population consisted of neoplasm-related deaths with death-certificate-documented protein-energy malnutrition among decedents aged ≥15 years. The primary outcomes were annual death counts and AAMR per 100,000 population. As a complementary denominator-based measure, we calculated the annual unadjusted proportion of all neoplasm-related deaths with documented protein-energy malnutrition. The numerator was the number of neoplasm-related deaths with ICD-10 E40–E46 recorded in the multiple-cause-of-death fields, and the denominator was the total number of neoplasm-related deaths in the same calendar year. Rates were calculated using the 2,000 US standard population, consistent with CDC WONDER settings (25). When available, 95% confidence intervals (CIs) and standard errors (SEs) were extracted directly from CDC WONDER. In accordance with CDC WONDER privacy and reliability standards, rates based on fewer than 10 deaths were suppressed.

### 2.3 Subgroup, category-specific, and state-level analyses

Temporal trends were examined across demographic and geographic strata, including sex, race, Hispanic origin, age group, census region, and urbanization. Sex was categorized as female or male. For descriptive race-specific analyses, race was grouped as White, Black, and Other; the latter included Asian, Native Hawaiian or Other Pacific Islander, and American Indian or Alaska Native groups combined because of small sample sizes in certain stratified analyses. Given the heterogeneity of this combined category, estimates for the other racial group were interpreted cautiously and were not intended to represent a single homogeneous population. Hispanic origin was analyzed independently of race and categorized as Hispanic or Non-Hispanic; records with origin not stated were not included in this supplementary subgroup analysis. The primary age grouping was 15–64 years and ≥65 years. To evaluate heterogeneity within the broad older-adult category, supplementary detailed age analyses used five groups: 15–44, 45–64, 65–74, 75–84, and ≥85 years. Census region was categorized as Northeast, Midwest, South, and West. Urbanization was categorized as metropolitan and nonmetropolitan, although comparable post-2020 urbanization data were unavailable; therefore, urban-rural analyses were restricted to 1999–2020. For neoplasm-category analyses, detailed UCD codes were grouped into clinically meaningful categories based on ICD-10 ranges: head and neck/oral cavity-pharynx (C00–C14), digestive organs (C15–C26), respiratory and intrathoracic organs (C30–C39), breast (C50), female genital organs (C51–C58), male genital organs (C60–C63), urinary tract (C64–C68), and lymphoid and hematopoietic tissue (C81–C96). Remaining codes, including D00–D48 and those outside these major groups, were classified as other neoplasms. For these broad neoplasm categories, annual observed death counts were reconstructed by summing mutually exclusive displayed detailed UCD cells within each calendar year. Category-specific AAMRs were reconstructed by summing component-specific age-adjusted rates within the same year, and standard errors were combined as an approximation assuming independence using the square root of the sum of squared standard errors; 95% CIs were then recalculated. Because CDC WONDER suppresses some small detailed UCD cells, reconstructed broad-category counts do not necessarily sum exactly to the national aggregate total. Accordingly, category-specific AAMRs and 95% CIs were interpreted as descriptive approximations, and no formal between-category comparisons were performed. To assess heterogeneity within the largest broad category, digestive neoplasms were further descriptively decomposed into colorectal/anus, pancreas, stomach, esophagus, liver/intrahepatic bile ducts, gallbladder/extrahepatic biliary tract, and other digestive sites. State-level analyses were performed for all US states and the District of Columbia from 1999 through 2024. Two state-level metrics were examined: the AAMR in 2024 and the average annual percent change (AAPC) from 1999 to 2024. In addition, to describe compositional shifts across broad neoplasm categories, we calculated the percentage contribution of each category among all observed displayed detailed neoplasm-category deaths in selected years (1999, 2005, 2010, 2015, 2020, and 2024).

### 2.4 Statistical analysis

Joinpoint trend analysis was performed using the Joinpoint Regression Program (Version 5.4.0.0; National Cancer Institute) (26). A log-linear model with up to two joinpoints was specified for the primary analysis to capture major macroscopic trend shifts while avoiding overfitting over the study period. Annual percent change (APC), AAPC, and corresponding 95% CIs were estimated. To assess temporal robustness, additional analyses allowed up to three joinpoints, restricted the observation period to pre-pandemic years (1999–2019), and restricted the analysis to the bridged-race dataset (1999–2020). To assess the potential influence of the data-system transition, overall, White, and Black AAMRs from bridged-race and single-race files were compared during the 2018–2020 overlap period; the heterogeneous Other racial category was not included in direct cross-system comparisons. To assess the robustness of the primary outcome definition, we conducted two additional sensitivity analyses. Sensitivity analysis A restricted the underlying cause of death to malignant neoplasms only (ICD-10 C00–C97) while retaining documented protein-energy malnutrition in the multiple-cause-of-death fields (ICD-10 E40–E46). Sensitivity analysis B used the same malignant-neoplasm restriction and expanded the multiple-cause definition to include either documented protein-energy malnutrition (E40–E46) or cachexia (R64). For the annual proportion of neoplasm-related deaths with documented protein-energy malnutrition, 95% confidence intervals were calculated using the Wilson method. The annual proportion was subsequently analyzed using the same log-linear Joinpoint framework and was interpreted as a denominator-based percentage rather than as an age-adjusted mortality rate. For subgroup and category-specific analyses, results were summarized as death counts and AAMRs for 1999 and 2024, together with overall AAPCs. Hispanic-origin analyses used AAMRs because age-adjusted rates were directly available from CDC WONDER. For detailed age-group analyses, we calculated age-specific mortality rates per 100,000 population by dividing annual deaths in each age group by the corresponding annual age-specific population; these rates were analyzed using the same Joinpoint framework and are reported separately from AAMRs. Statistical significance for trend estimates was determined using the permutation test implemented in the Joinpoint software, and a two-sided *P* < 0.05 was considered statistically significant. Given the descriptive and exploratory nature of the multiple subgroup and state-level trend analyses, *P* values were interpreted cautiously and were not adjusted for multiple comparisons. All other statistical analyses and figure generation were conducted in R.

## 3 Results

### 3.1 Overall burden and temporal trends

**Figure 1:**
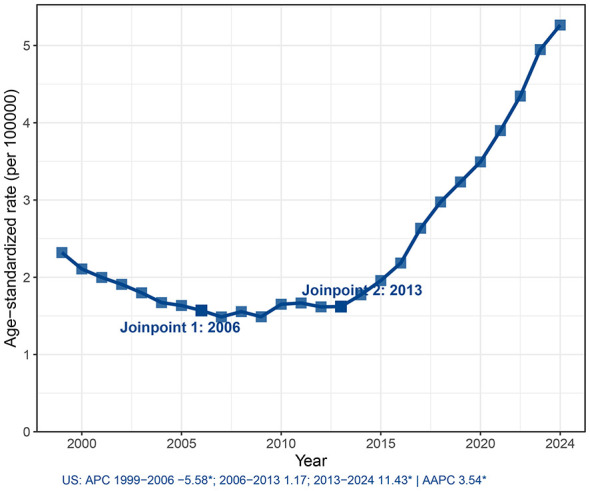
Overall temporal trends in AAMR for neoplasm-related deaths with death-certificate-documented protein-energy malnutrition in the United States, 1999–2024. AAMR, age-adjusted mortality rate; AAPC, average annual percent change; APC, annual percent change.

Between 1999 and 2024, the annual number of neoplasm-related deaths with death-certificate-documented protein-energy malnutrition increased from 5,024 to 18,410. Over the same period, the AAMR increased from 2.32 (95% CI, 2.26–2.38) to 5.26 (95% CI, 5.19–5.34) per 100,000 population (Table 1). Joinpoint analysis identified two inflection points in 2006 and 2013. The AAMR declined significantly from 1999 to 2006 (APC, −5.58%; 95% CI, −9.84% to −3.88%), changed little from 2006 to 2013 (APC, 1.17%; 95% CI, −1.29% to 5.63%), and then rose sharply from 2013 to 2024 (APC, 11.43%; 95% CI, 10.80% to 12.32%), yielding an overall AAPC of 3.54% (95% CI, 3.28% to 3.90%) (Figure 1).

To assess whether the increase reflected only changes in the total number of neoplasm-related deaths, we examined the annual proportion of all neoplasm-related deaths with documented protein-energy malnutrition. This proportion increased from 0.90% (5,024 of 561,336 neoplasm-related deaths; 95% CI, 0.87%−0.92%) in 1999 to 2.90% (18,410 of 634,842 deaths; 95% CI, 2.86%−2.94%) in 2024. Joinpoint analysis showed a decline from 1999 to 2007 (APC, −3.76%; 95% CI, −6.93% to −2.27%), a modest increase from 2007 to 2013 (APC, 3.81%; 95% CI, 0.13% to 8.79%), and a marked increase from 2013 to 2024 (APC, 12.92%; 95% CI, 12.24% to 13.95%). The overall AAPC was 5.14% (95% CI, 4.86% to 5.50%) (Supplementary Table S1; Supplementary Figure S1).

Sensitivity analyses showed that the main temporal pattern was robust to alternative outcome definitions. When the analysis was restricted to malignant neoplasms only (C00–C97) with documented protein-energy malnutrition (E40–E46), deaths increased from 4,909 in 1999 to 17,915 in 2024, while the AAMR increased from 2.28 (95% CI, 2.22–2.35) to 5.11 (95% CI, 5.04–5.19) per 100,000 population. Under this strict malignant-neoplasm definition, the AAMR declined from 1999 to 2007, remained statistically stable from 2007 to 2013, and increased markedly from 2013 to 2024 (APC, 11.34%; 95% CI, 10.83%−12.03%), with an overall AAPC of 3.56% (95% CI, 3.35%−3.83%). When the definition was expanded to include either documented protein-energy malnutrition or cachexia (R64), deaths increased from 8,698 in 1999 to 19,756 in 2024 and the AAMR increased from 4.06 (95% CI, 3.97–4.14) to 5.65 (95% CI, 5.57–5.73) per 100,000 population. This expanded definition also showed a significant late-period increase from 2014 to 2024 (APC, 8.64%; 95% CI, 8.11%−9.38%) (Supplementary Table S2; Supplementary Figure S2).

Temporal robustness analyses showed that the late-period increase was not materially altered by model flexibility, exclusion of pandemic years, or restriction to the bridged-race data system. When up to three joinpoints were permitted, the overall AAPC was 3.51% (95% CI, 3.29% to 3.81%), with significant increases during 2014–2018 (APC, 14.20%; 95% CI, 2.21% to 17.90%) and 2018–2024 (APC, 10.44%; 95% CI, 8.69% to 11.61%). In the pre-pandemic analysis restricted to 1999–2019, the final increasing segment persisted from 2014 to 2019 (APC, 13.82%; 95% CI, 11.31% to 20.51%). Similarly, in the bridged-race-only analysis restricted to 1999–2020, AAMRs increased significantly from 2013 to 2020 (APC, 12.03%; 95% CI, 10.74% to 14.36%). During the 2018–2020 overlap period, overall AAMRs were virtually identical between bridged-race and single-race files, whereas White and Black estimates differed only modestly and had overlapping 95% CIs in every comparison (Supplementary Table S3; Supplementary Figure S3).

### 3.2 Demographic disparities

**Figure 2:**
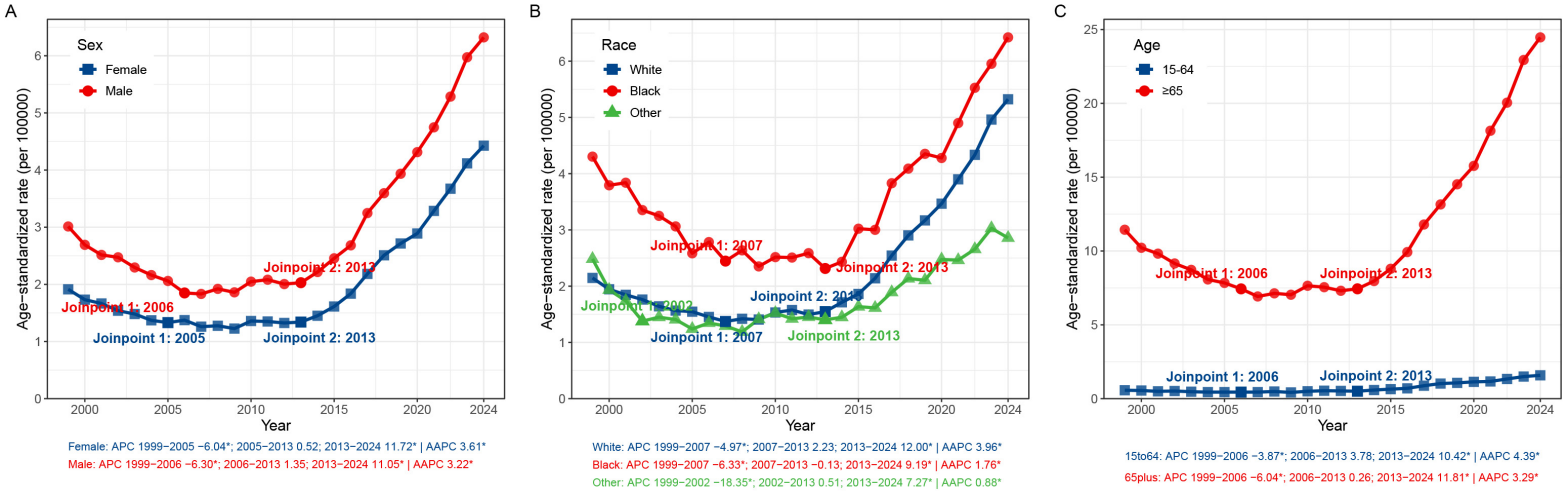
Trends in AAMR for neoplasm-related deaths with death-certificate-documented protein-energy malnutrition by demographic characteristics in the United States, 1999–2024. **(A)** Sex. **(B)** Race. **(C)** Age group. AAMR, age-adjusted mortality rate; AAPC, average annual percent change; APC, annual percent change.

Clear sex differences were observed throughout the study period. In 1999, the AAMR was higher among males than females (3.01 vs. 1.91 per 100,000), and this gap persisted in 2024 (6.32 vs. 4.43 per 100,000). Both sexes showed an early decline followed by an accelerated rise after 2013; however, the overall long-term increase was slightly greater among females than males (AAPC, 3.61% vs. 3.22%) (Table 1; Figure 2A).

**Table 1:** Overall and subgroup trends in neoplasm-related mortality with death-certificate-documented protein-energy malnutrition, United States, 1999–2024.

Categories	1999		2024		AAPC (95% CI)
	Counts	AAMR (95% CI)	Counts	AAMR (95% CI)	
US total	5,024	2.32 (2.26, 2.38)	18,410	5.26 (5.19, 5.34)	3.54 (3.28 to 3.90)^*^
Sex					
Female	2,443	1.91 (1.84, 1.99)	8,436	4.43 (4.33, 4.52)	3.61 (3.35 to 3.92)^*^
Male	2,581	3.01 (2.89, 3.13)	9,974	6.32 (6.20, 6.45)	3.22 (2.96 to 3.60)^*^
Age					
≥65	3,962	11.44 (11.08, 11.80)	14,264	24.47 (24.07, 24.88)	3.29 (3.05 to 3.63)^*^
15–64	1,062	0.57 (0.54, 0.61)	4,146	1.58 (1.53, 1.63)	4.39 (3.86 to 5.07)^*^
Race					
Black	786	4.30 (4.00, 4.61)	2,386	6.42 (6.16, 6.69)	1.76 (1.38 to 2.20)^*^
Other	143	2.48 (2.05, 2.92)	792	2.86 (2.66, 3.07)	0.88 (0.37 to 1.67)^*^
White	4,095	2.15 (2.08, 2.21)	15,232	5.32 (5.24, 5.41)	3.96 (3.75 to 4.22)^*^
Urbanization^a^					
Metropolitan	3,786	2.18 (2.11, 2.25)	9,432	3.41 (3.34, 3.48)	2.46 (2.08 to 2.94)^*^
Nonmetropolitan	1,238	3.07 (2.90, 3.24)	2,150	4.03 (3.85, 4.21)	1.50 (1.17 to 1.85)^*^
Census region					
Northeast	771	1.70 (1.58, 1.82)	2,069	3.19 (3.05, 3.34)	2.72 (2.46 to 3.05)^*^
Midwest	1,389	2.70 (2.56, 2.84)	4,315	5.88 (5.71, 6.07)	3.34 (3.06 to 3.65)^*^
South	1,916	2.54 (2.42, 2.65)	7,448	5.57 (5.45, 5.70)	3.30 (2.91 to 3.70)^*^
West	948	2.23 (2.09, 2.37)	4,578	5.81 (5.64, 5.98)	4.47 (4.07 to 5.05)^*^

AAMR, age-adjusted mortality rate; AAPC, average annual percent change; CI, confidence interval. Urbanization data were available only for 1999–2020. For metropolitan and nonmetropolitan areas, the values presented under the 2024 columns represent estimates for 2020, and the corresponding AAPCs were calculated for 1999–2020.

^*^Indicates a statistically significant trend (*P* < 0.05).

Marked racial disparities were also present. Black individuals had the highest AAMR at both the beginning and end of the study period, increasing from 4.30 in 1999 to 6.42 per 100,000 in 2024. The corresponding AAMRs increased from 2.15 to 5.32 among White individuals and from 2.48 to 2.86 among individuals in the “Other” racial category. Despite the persistently higher absolute burden among Black individuals, the steepest overall increase was observed among White individuals (AAPC, 3.96%), followed by Black individuals (1.76%) and the “Other” group (0.88%) (Table 1; Figure 2B). Figure 2B further shows that all racial groups experienced a renewed upward trajectory after 2013, although the magnitude of increase was greatest for White individuals.

In supplementary analyses by Hispanic origin, AAMRs increased in both Hispanic and Non-Hispanic populations. Among Hispanic decedents, the AAMR increased from 1.97 (95% CI, 1.69–2.25) per 100,000 in 1999 to 3.78 (95% CI, 3.59–3.99) in 2024, with an overall AAPC of 3.04% (95% CI, 2.43%−3.95%). Among Non-Hispanic decedents, the AAMR increased from 2.37 (95% CI, 2.30–2.44) to 5.42 (95% CI, 5.34–5.50) per 100,000, with an AAPC of 3.62% (95% CI, 3.37%−3.94%). Thus, rates remained lower among Hispanic than Non-Hispanic decedents at both time points, although both groups experienced significant long-term increases (Supplementary Table S4; Supplementary Figure S4A).

Age-stratified analyses showed that the burden was concentrated among older adults but varied substantially within the broad ≥65-year category. In the primary two-group analysis, the AAMR among adults aged ≥65 years increased from 11.44 in 1999 to 24.47 per 100,000 in 2024, whereas among those aged 15–64 years it increased from 0.57 to 1.58 per 100,000. Although the younger group had a higher overall AAPC than the older group (4.39% vs. 3.29%), the absolute mortality burden remained substantially greater among older adults throughout the study period (Figure 2C; Table 1). In the detailed age analysis, 2024 age-specific mortality rates increased stepwise with age, from 0.29 per 100,000 among persons aged 15–44 years to 4.54 among those aged 45–64 years, 15.30 among those aged 65–74 years, 28.02 among those aged 75–84 years, and 53.33 among those aged ≥85 years. All five detailed age groups showed significant long-term increases. The largest relative increases were observed among adults aged 15–44 years and 45–64 years, with AAPCs of 4.94% and 4.93%, respectively, whereas the absolute mortality burden remained greatest among adults aged ≥85 years (Supplementary Table S4; Supplementary Figure S4B).

### 3.3 Geographic disparities

**Figure 3:**
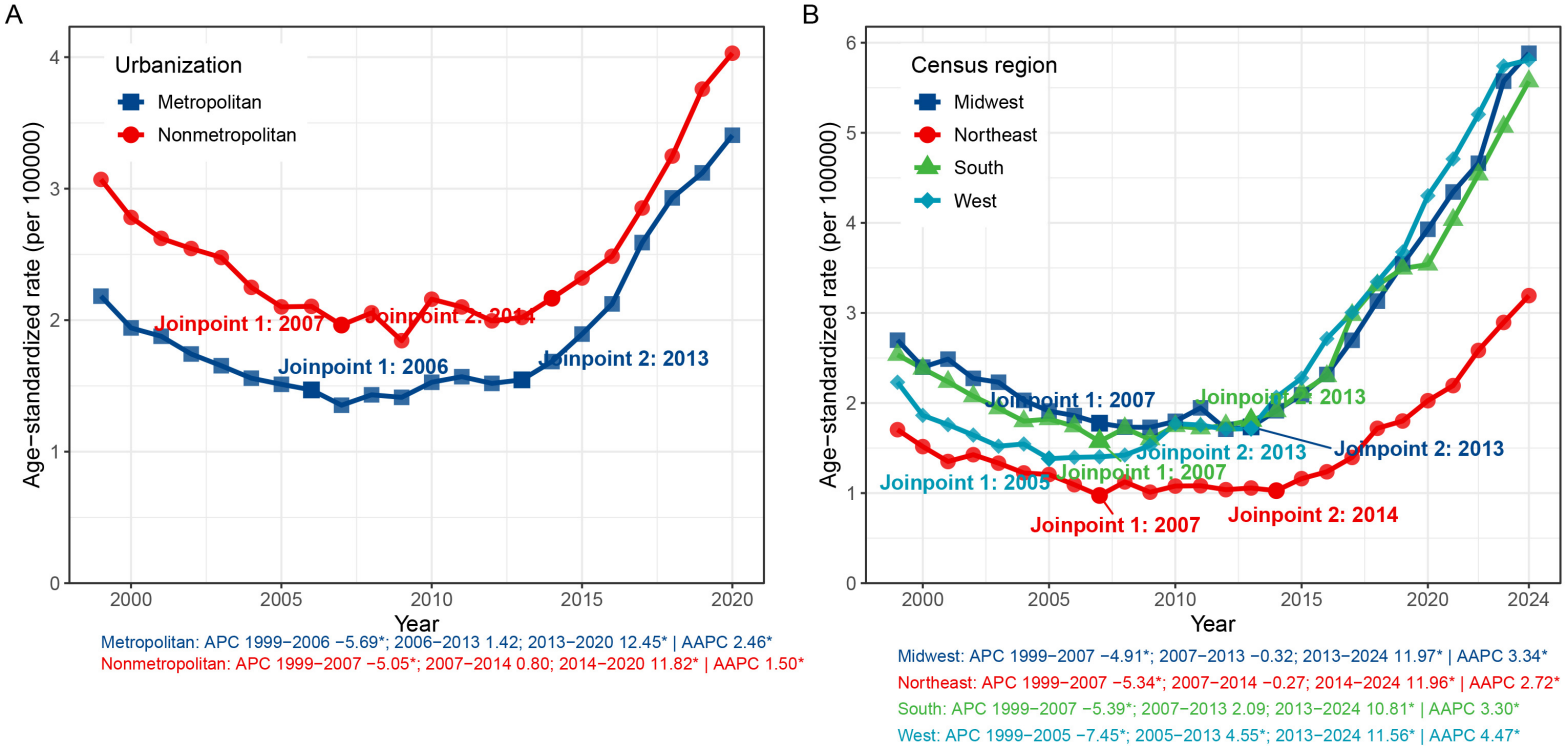
Trends in age-adjusted mortality rates for neoplasm-related deaths with death-certificate-documented protein-energy malnutrition by geographic characteristics in the United States. **(A)** Urbanization trends, 1999–2020. **(B)** Census-region trends, 1999–2024. AAMR, age-adjusted mortality rate; AAPC, average annual percent change; APC, annual percent change.

Urban-rural analyses, limited to 1999–2020, showed consistently higher mortality in nonmetropolitan than metropolitan areas. The AAMR in nonmetropolitan areas increased from 3.07 in 1999 to 4.03 per 100,000 in 2020, whereas the corresponding increase in metropolitan areas was from 2.18 to 3.41 per 100,000. Both strata followed a similar pattern of early decline followed by later increase, but the long-term AAPC was somewhat higher in metropolitan than nonmetropolitan areas (2.46% vs. 1.50%) (Table 1; Figure 3A).

Substantial regional variation was also evident. In 1999, the Midwest had the highest AAMR (2.70 per 100,000), followed by the South (2.54), West (2.23), and Northeast (1.70). By 2024, the Midwest and West had the highest AAMRs (5.88 and 5.81 per 100,000, respectively), followed by the South (5.57), whereas the Northeast remained markedly lower at 3.19 per 100,000. The West had the largest overall increase (AAPC, 4.47%), followed by the Midwest (3.34%), South (3.30%), and Northeast (2.72%) (Table 1; Figure 3B).

### 3.4 State-level variation

**Figure 4:**
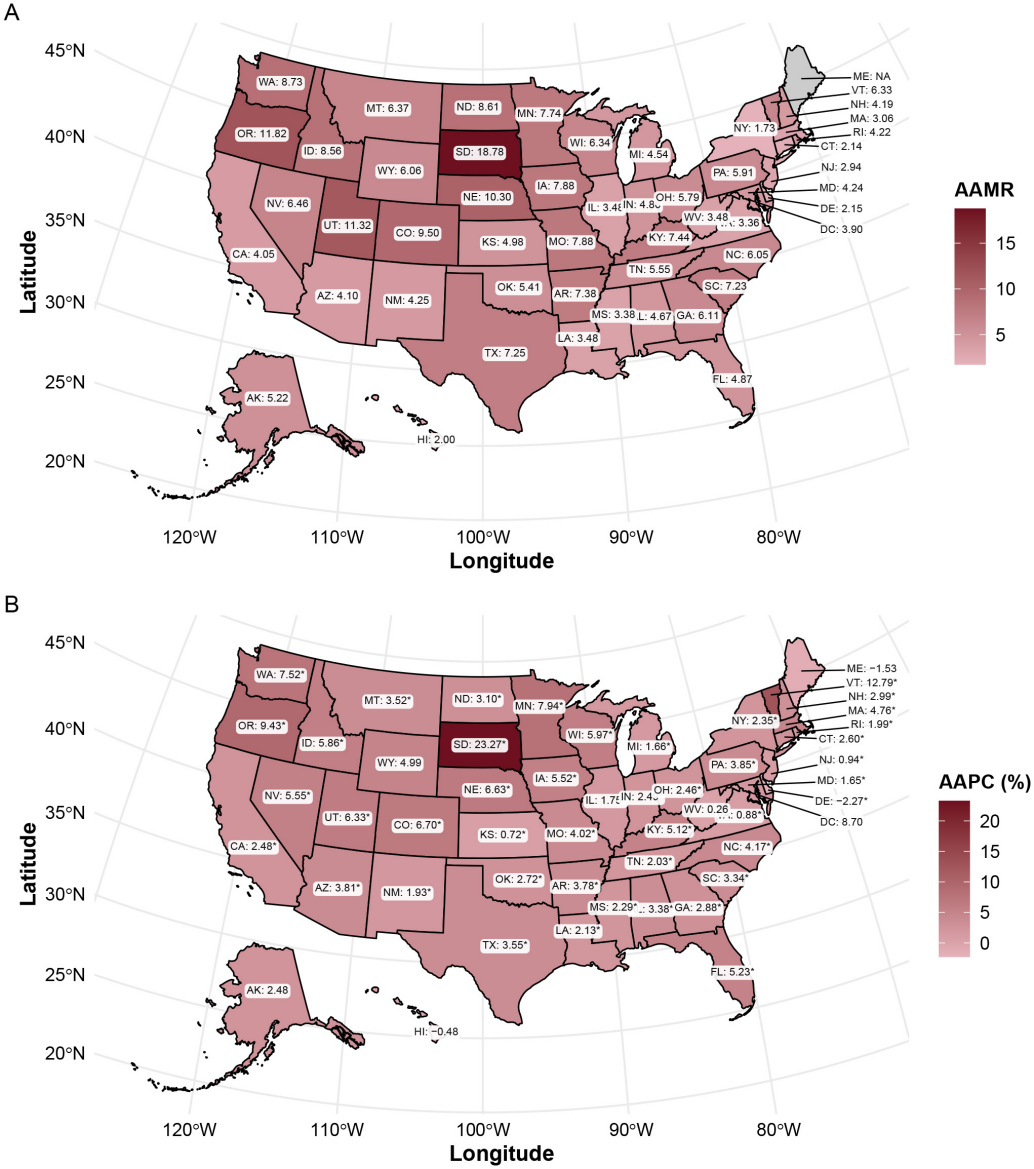
State-level heterogeneity in mortality burden and temporal trends for neoplasm-related deaths with death-certificate-documented protein-energy malnutrition in the United States, 1999–2024. **(A)** AAMR in 2024. **(B)** AAPC during 1999–2024. AAMR, age-adjusted mortality rate; AAPC, average annual percent change; APC, annual percent change.

State-level analyses revealed substantial geographic heterogeneity in both contemporary burden and long-term temporal change. In 2024, the AAMR ranged from 1.73 per 100,000 in New York to 18.78 per 100,000 in South Dakota. Other states with particularly high 2024 AAMRs included Oregon (11.82), Utah (11.32), Nebraska (10.30), and Colorado (9.50), whereas relatively low AAMRs were observed in New York (1.73), Hawaii (2.00), Connecticut (2.14), and Delaware (2.15) (Supplementary Table S5; Figure 4A).

Long-term AAPCs also varied widely across states. The highest AAPCs were observed in South Dakota (23.27%), Vermont (12.79%), Oregon (9.43%), Minnesota (7.94%), and Washington (7.52%). Delaware was the only state with a significant negative AAPC (−2.27%), whereas Maine (−1.53%) and Hawaii (−0.48%) showed non-significant declines. Overall, Figure 4B demonstrates marked state-level heterogeneity in the pace of increase in neoplasm-related mortality with death-certificate-documented protein-energy malnutrition. Because state-specific estimates may be unstable where counts are sparse, particularly in small-population jurisdictions, state-level AAPCs should be interpreted cautiously. Corresponding uncertainty estimates are presented in Supplementary Table S5 where available.

### 3.5 Variation across neoplasm categories

Pronounced heterogeneity was observed across broad neoplasm categories. In 1999, digestive neoplasms accounted for the largest observed category-specific burden, with 1,598 observed deaths and an approximate reconstructed AAMR of 0.65 (95% CI, 0.61–0.68) per 100,000, followed by respiratory and intrathoracic neoplasms [936 observed deaths; approximate reconstructed AAMR, 0.42 (95% CI, 0.39–0.45)] and other neoplasms [683 observed deaths; approximate reconstructed AAMR, 0.24 (95% CI, 0.22–0.27)]. By 2024, these same categories remained the largest observed contributors, with digestive neoplasms contributing 5,702 deaths [approximate reconstructed AAMR, 1.50 (95% CI, 1.45–1.54)], respiratory and intrathoracic neoplasms contributing 3,167 deaths [approximate reconstructed AAMR, 0.86 (95% CI, 0.83–0.89)], and other neoplasms contributing 3,012 deaths [approximate reconstructed AAMR, 0.65 (95% CI, 0.61–0.68)] (Table 2). Because broad-category estimates were reconstructed from displayed detailed UCD cells, category counts did not sum exactly to the national aggregate total. Displayed detailed categories represented 4,662 of 5,024 national deaths (92.8%) in 1999 and 17,745 of 18,410 national deaths (96.4%) in 2024 (Supplementary Table S6; Supplementary Figure S5A).

**Table 2:** Descriptive broad neoplasm-category patterns among neoplasm-related deaths with death-certificate-documented protein-energy malnutrition in the United States, 1999–2024.

Neoplasm categories	1999		2024		AAPC (95% CI)
	Observed deaths reconstructed from displayed detailed UCD cells, *n*	Approximate reconstructed AAMR per 100,000 population (95% CI)	Observed deaths reconstructed from displayed detailed UCD cells, *n*	Approximate reconstructed AAMR per 100,000 population (95% CI)	
Breast	246	0.12 (0.10, 0.13)	1,058	0.31 (0.29, 0.33)	4.83 (4.23 to 5.70)^*^
Digestive organs	1,598	0.65 (0.61, 0.68)	5,702	1.50 (1.45, 1.54)	3.58 (3.19 to 4.09)^*^
Female genital	257	0.08 (0.07, 0.09)	862	0.20 (0.18, 0.22)	3.88 (3.07 to 4.95)^*^
Head and neck/oral cavity-pharynx	151	0.04 (0.03, 0.05)	644	0.14 (0.13, 0.15)	6.04 (4.24 to 9.16)^*^
Lymphoid and hematopoietic tissue	313	0.10 (0.08, 0.12)	1,320	0.32 (0.30, 0.34)	4.76 (4.13 to 5.60)^*^
Male genital	294	0.14 (0.12, 0.15)	1,077	0.30 (0.28, 0.31)	3.22 (2.72 to 3.99)^*^
Other neoplasms	683	0.24 (0.22, 0.27)	3,012	0.65 (0.61, 0.68)	4.45 (3.80 to 5.33)^*^
Respiratory and intrathoracic organs	936	0.42 (0.39, 0.45)	3,167	0.86 (0.83, 0.89)	2.96 (2.53 to 3.52)^*^
Urinary	184	0.07 (0.06, 0.08)	903	0.25 (0.23, 0.27)	5.44 (4.72 to 6.53)^*^

AAMR, age-adjusted mortality rate; AAPC, average annual percent change; CI, confidence interval; UCD, underlying cause of death.

^*^Indicates a statistically significant trend (*P* < 0.05). Category-specific counts were reconstructed from displayed detailed UCD ICD-10 cells. Because CDC WONDER suppresses some small detailed cells, category-specific counts do not necessarily sum exactly to the national aggregate total. Category-specific AAMRs and 95% CIs were reconstructed from mutually exclusive displayed detailed codes and should be interpreted as descriptive approximations. No formal between-category comparisons were performed.

Descriptively, the largest AAPC point estimate was observed for head and neck/oral cavity-pharynx neoplasms (6.04%), followed by urinary tract neoplasms (5.44%), breast neoplasms (4.83%), lymphoid and hematopoietic tissue neoplasms (4.76%), and other neoplasms (4.45%). Digestive neoplasms showed a moderate but sustained long-term increase (AAPC, 3.58%), whereas respiratory and intrathoracic neoplasms had the smallest AAPC point estimate among the broad categories presented (2.96%) (Table 2). Segment-specific APC estimates showed that most major categories shared a common pattern of decline in the early years followed by marked increases after approximately 2013, particularly for digestive, respiratory, breast, female genital, hematologic, and other neoplasms (Supplementary Table S7).

**Figure 5:**
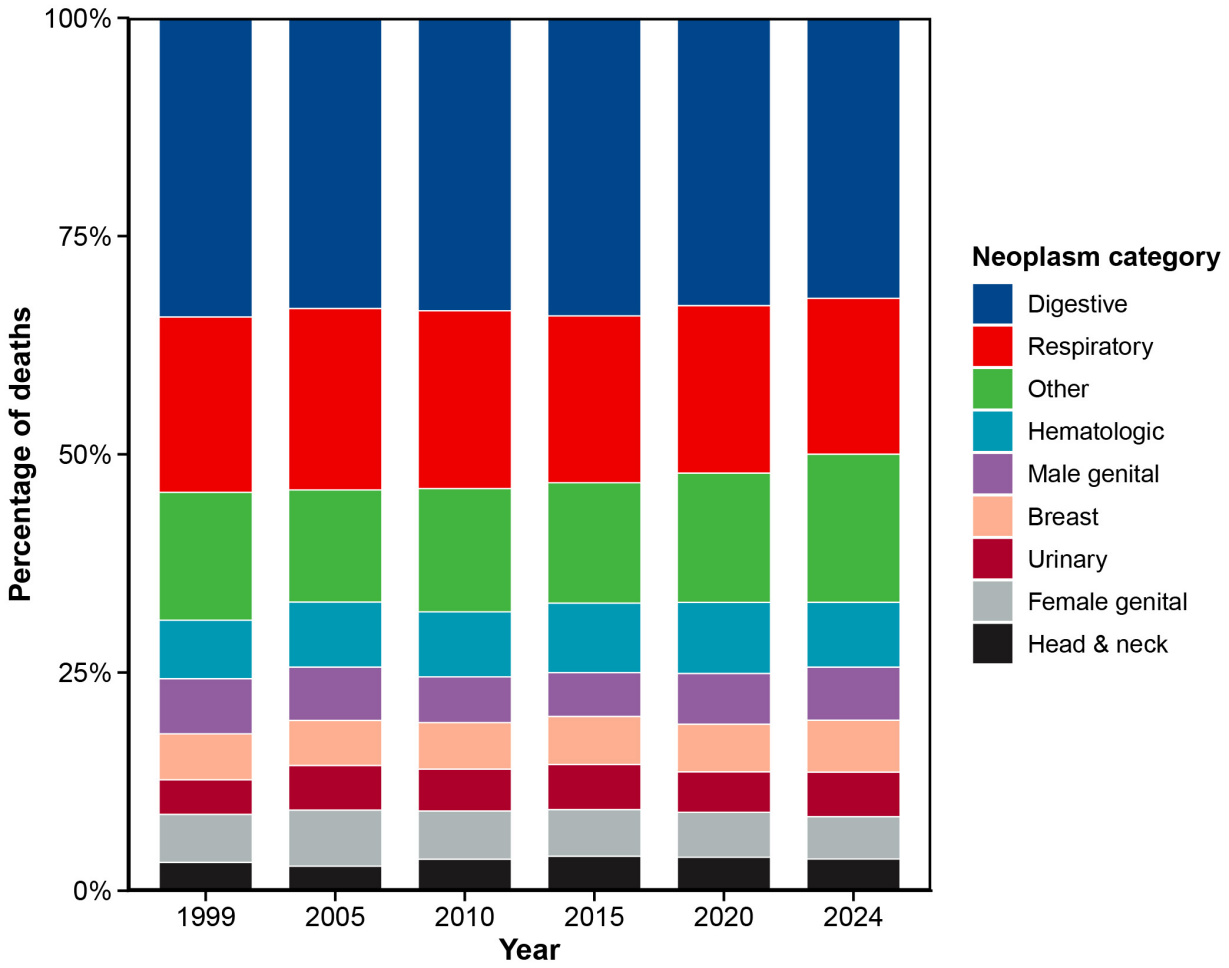
Changes in the proportional distribution of observed detailed broad neoplasm-category deaths with death-certificate-documented protein-energy malnutrition in the United States across selected years, 1999–2024. Percentages were calculated using the sum of displayed detailed UCD-category deaths in each selected year as the denominator.

The proportional composition of observed detailed neoplasm-category deaths remained broadly stable over time, although several shifts were apparent in selected years. Digestive neoplasms consistently represented the largest observed category, followed by respiratory and intrathoracic neoplasms. Among displayed detailed category deaths, digestive neoplasms accounted for 34.3% in 1999 and 32.1% in 2024, respiratory and intrathoracic neoplasms accounted for 20.1% and 17.8%, and other neoplasms accounted for 14.7% and 17.0%, respectively. These proportions were calculated using the sum of displayed detailed category deaths as the denominator and therefore should not be interpreted as exact proportions of all national neoplasm-related deaths (Figure 5; Supplementary Table S6).

Within digestive neoplasms, the burden was heterogeneous rather than evenly distributed. In 2024, the largest observed digestive subsites were colorectal/anus neoplasms (1,841 deaths; 32.3% of observed digestive-category deaths), pancreatic neoplasms (1,421 deaths; 24.9%), esophageal neoplasms (941 deaths; 16.5%), liver and intrahepatic bile duct neoplasms (678 deaths; 11.9%), and stomach neoplasms (520 deaths; 9.1%). Thus, the broad digestive category was primarily driven by a limited number of subsites. These results should be interpreted as descriptive decompositions of displayed detailed UCD counts rather than suppression-free site-specific burden estimates or formal site-specific temporal trend analyses (Supplementary Table S6; Supplementary Figure S5B).

## 4 Discussion

In this national study of neoplasm-related deaths with death-certificate-documented protein-energy malnutrition in the United States, we found that the mortality burden increased substantially between 1999 and 2024. Annual deaths rose from 5,024 to 18,410, and the AAMR increased from 2.32 to 5.26 per 100,000 population, with an overall AAPC of 3.54%. Importantly, the increase was also evident when the outcome was expressed as a proportion of all neoplasm-related deaths, which rose from 0.90% to 2.90% over the study period. This increase was not linear. Instead, the overall trend was characterized by an early decline, a relatively stable intermediate period, and a marked increase after approximately 2013. In addition, the burden was not evenly distributed across the population: clear disparities were observed by sex, race, age, urbanization, census region, neoplasm category, and state. Together, these findings indicate that death-certificate-documented protein-energy malnutrition has become an increasingly prominent feature of neoplasm-related mortality in the United States and that this burden is concentrated in specific demographic, geographic, and disease-defined subgroups.

The temporal pattern observed in this study warrants particular attention, but it should be interpreted cautiously. The overall AAMR declined from 1999 to 2006, changed little from 2006 to 2013, and then rose sharply thereafter. The pattern was retained when the analysis was restricted to malignant neoplasms only, indicating that the primary findings were not driven by the inclusion of *in situ*, benign, or behavior-uncertain neoplasms within the broader C00–D48 definition. In addition, the late-period increase persisted after the outcome definition was expanded to include cachexia (R64), although the final inflection occurred in 2014 rather than 2013 and the overall long-term AAPC was smaller under the expanded definition. These findings suggest that the post-2013/post-2014 increase was not dependent on a single narrow coding definition. COVID-19 may have altered cancer care access and death-certificate completion during 2020–2021. However, the pre-pandemic sensitivity analysis ending in 2019 retained a marked final increasing segment from 2014 to 2019 (APC, 13.82%; 95% CI, 11.31%−20.51%), indicating that pandemic-era observations alone cannot account for the post-2013 trajectory. We did not identify a discrete national coding or certification update specific to ICD-10 E40–E46 around 2006 or 2013 that would justify attributing this data-driven inflection point to a single coding or certification change.

Nevertheless, the present analyses do not establish that the biological prevalence of protein-energy malnutrition or cachexia increased to the same extent. Population aging, increasing survival with advanced disease, and prolonged exposure to multimodality cancer treatment may have increased the burden of severe nutritional vulnerability among decedents with neoplasms (27–30). At the same time, changes in clinical recognition, nutritional assessment, death-certificate completion, and coding practices may also have contributed to the observed temporal pattern (19, 31–33). The denominator-based analysis indicates that the increase was not explained solely by growth in the total number of neoplasm-related deaths; however, this finding should not be interpreted as direct evidence that the biological prevalence of protein-energy malnutrition increased to the same extent. Because death-certificate-documented protein-energy malnutrition likely represents a severe and clinically recognized subset of nutritional compromise, the observed trends may reflect changes in both underlying burden and documentation practice (21, 33). This distinction remains important when comparing the present findings with clinical studies that assess malnutrition using formal nutritional screening tools, anthropometric measures, or cachexia-related definitions.

The subgroup analyses revealed substantial demographic heterogeneity. Male decedents consistently had higher AAMRs than female decedents, although the overall long-term increase was slightly greater among females. Black individuals experienced the highest AAMRs at both the beginning and end of the study period, whereas White individuals showed the steepest overall increase. In supplementary analyses, both Hispanic and Non-Hispanic populations experienced significant long-term increases, although AAMRs were lower among Hispanic decedents at both endpoints. These findings suggest that the burden of death-certificate-documented protein-energy malnutrition in neoplasm-related mortality remains unequally distributed and may be shaped by differences in neoplasm profile, comorbidity burden, healthcare access, supportive care utilization, and broader structural inequities (34–36). Age differences were especially marked. The detailed age analysis showed a steep gradient within the older population, with the 2024 age-specific mortality rate increasing from 15.30 per 100,000 among adults aged 65–74 years to 28.02 among those aged 75–84 years and 53.33 among those aged ≥85 years. At the same time, the relative long-term increase was greatest among adults aged 15–44 years and 45–64 years. Thus, older adults remained the highest-burden population, while younger and middle-aged adults showed the fastest proportional growth in death-certificate-documented protein-energy malnutrition. The high absolute burden among older adults is clinically plausible given the greater prevalence of frailty, sarcopenia, multimorbidity, functional impairment, and treatment intolerance among older adults with neoplasms (30, 37–39).

Geographic disparities were also pronounced. Nonmetropolitan areas had consistently higher AAMRs than metropolitan areas during 1999–2020, indicating a persistent rural disadvantage in the burden of death-certificate-documented protein-energy malnutrition among neoplasm-related deaths (40, 41). At the regional level, the Midwest had the highest AAMR in 1999, while by 2024 both the Midwest and West had the highest rates, and the West showed the steepest overall increase. These findings may reflect regional differences in population structure, neoplasm mix, healthcare access, nutritional support resources, and palliative or supportive care capacity (2, 34, 41). They may also reflect differences in the clinical recognition and death-certificate documentation of protein-energy malnutrition (2, 34). Although the present data cannot distinguish among these mechanisms, the consistency of the regional patterns suggests that malnutrition-related vulnerability at the end of life is shaped, at least in part, by broader contextual factors beyond individual clinical characteristics.

The state-level analyses further extended these observations by demonstrating striking spatial heterogeneity across the United States. In 2024, the AAMR ranged from 1.73 per 100,000 in New York to 18.78 per 100,000 in South Dakota, with particularly high values also observed in Oregon, Utah, Nebraska, and Colorado. Long-term AAPCs likewise varied considerably, reaching 23.27% in South Dakota and exceeding 7% in Vermont, Oregon, Minnesota, and Washington, whereas Delaware showed a significant negative AAPC. These state-level contrasts are unlikely to be explained by a single factor. Rather, they may reflect differences in rurality, state population structure, underlying neoplasm distributions, access to oncology and nutrition services, healthcare delivery systems, and local documentation practices (41–45). From a public health perspective, these findings suggest that nutritional vulnerability among people dying from neoplasms is not distributed uniformly across states and that targeted surveillance and supportive care strategies may be warranted in high-burden jurisdictions (41, 43, 45).

Substantial heterogeneity was also evident across broad neoplasm categories. Digestive neoplasms contributed the largest observed broad-category burden in both 1999 and 2024 and consistently accounted for the largest observed share of detailed category deaths. However, the digestive category should not be interpreted as homogeneous. The descriptive subsite decomposition showed that colorectal/anus and pancreatic neoplasms together accounted for more than half of observed digestive-category deaths with documented protein-energy malnutrition in 2024, while esophageal, liver/intrahepatic bile duct, and stomach neoplasms also contributed substantially. These patterns are clinically coherent because digestive neoplasms may impair oral intake, swallowing, digestion, absorption, and metabolic homeostasis and are often associated with weight loss, cachexia, obstruction, nausea, or malabsorption (46). Respiratory and intrathoracic neoplasms were the second largest observed broad-category contributor and are commonly associated with systemic inflammatory catabolism and advanced disease (47). In contrast, the steepest long-term increases were observed for head and neck, urinary tract, breast, hematologic, and other neoplasms. For head and neck neoplasms in particular, compromised swallowing, treatment-related mucosal toxicity, and prolonged feeding difficulties may help explain the high AAPC despite relatively low absolute counts. More broadly, the increasing observed contribution of other neoplasms and hematologic neoplasms suggests that death-certificate-documented protein-energy malnutrition is not confined to traditionally recognized gastrointestinal or thoracic malignancies, but may be recorded across a wider range of neoplastic conditions (48, 49). Because broad-category and digestive-subsite estimates were reconstructed from displayed detailed UCD cells, these findings should be interpreted as descriptive rather than as suppression-free comparative estimates.

A major strength of this study is that it leveraged a large national mortality database over more than two decades to provide a comprehensive population-level assessment of death-certificate-documented protein-energy malnutrition among neoplasm-related deaths through integrated burden, trend, compositional, sensitivity, and state-level analyses. Several limitations should be considered. First, death certificate data likely underestimate the true prevalence of malnutrition, particularly milder forms that may not be clinically recognized or recorded; thus, the present study most likely reflects severe or explicitly documented nutritional vulnerability near the end of life. Second, the primary underlying cause-of-death definition included ICD-10 codes C00–D48 and therefore captured neoplasm-related mortality rather than strictly malignant neoplasm mortality only. However, the malignant-neoplasm-only sensitivity analysis restricted to C00–C97 showed a highly similar temporal pattern. Third, ICD-10 E40–E46 identifies death-certificate-documented protein-energy malnutrition rather than the full spectrum of nutritional deterioration, including cachexia, sarcopenia, and unintentional weight loss. Although the cachexia-inclusive sensitivity analysis yielded a higher absolute mortality burden and a somewhat smaller overall AAPC, it retained a significant late-period increase, supporting the robustness of the principal temporal finding across alternative coding definitions. Accordingly, the present results should be interpreted as trends in death-certificate-documented protein-energy malnutrition, with cachexia included only in a sensitivity analysis, rather than direct estimates of all forms of cancer-associated nutritional compromise. Fourth, the database does not provide individual-level clinical variables such as tumor stage, treatment history, weight loss trajectory, body mass index, cachexia severity, performance status, or nutritional interventions. Fifth, this was a descriptive mortality analysis and cannot support causal inference. Sixth, analyses combining 1999–2020 bridged-race data with 2021–2024 single-race data may be affected by differences in race classification and population denominators. However, the bridged-race-only analysis, pre-pandemic analysis, and 2018–2020 overlap comparisons did not indicate a material discontinuity in the overall temporal pattern. Direct overlap-period comparison was limited to Overall, White, and Black populations; the heterogeneous Other racial category was not considered directly comparable across the two data systems. Seventh, urban-rural analyses were limited to 1999–2020 because comparable post-2020 urbanization data were unavailable in the downloaded dataset. Finally, for broad neoplasm-category analyses, category-level counts, AAMRs, and confidence intervals were reconstructed from mutually exclusive displayed detailed UCD codes. Because CDC WONDER suppresses some small detailed cells, reconstructed category counts did not sum exactly to the national aggregate total, and category-specific AAMRs and confidence intervals should be interpreted as descriptive approximations. Similarly, digestive-subsite analyses were descriptive decompositions of displayed detailed UCD counts and were not intended to provide suppression-free site-specific burden estimates or formal site-specific temporal trend analyses.

## 5 Conclusion

In conclusion, death-certificate-documented protein-energy malnutrition among neoplasm-related deaths in the United States increased substantially from 1999 to 2024 and exhibited marked disparities by sex, race, age, geography, neoplasm category, and state. The principal late-period increase persisted in bridged-race-only, pre-pandemic, and flexible-joinpoint sensitivity analyses, suggesting that the overall temporal pattern was not materially altered by the transition between CDC WONDER data systems or by pandemic-era observations alone. The increase persisted when evaluated as a proportion of all neoplasm-related deaths, suggesting that it was not explained solely by changes in the overall volume of neoplasm-related mortality. The burden remained especially high among the oldest adults and Black individuals, while nonmetropolitan areas had higher rates than metropolitan areas during 1999–2020. These descriptive mortality data cannot distinguish changes in underlying nutritional vulnerability from changes in clinical recognition and death-certificate documentation. Nevertheless, the findings support greater attention to documented protein-energy malnutrition in oncology and supportive care pathways, alongside further studies that can disentangle changes in nutritional status from changes in documentation practices.

## Data Availability

Publicly available datasets were analyzed in this study. These data can be found in the CDC WONDER Multiple Cause of Death database (https://wonder.cdc.gov/). The processed results supporting the conclusions of this article are included in the article/Supplementary material; further inquiries can be directed to the corresponding author.
