# Supplementary material for: Trends and disparities in neoplasm-related mortality with death-certificate-documented protein-energy malnutrition in the United States, 1999–2024: a CDC WONDER multiple cause of death study
Source: Front Nutr. 2026 Jul 28;13:1860679. doi: 10.3389/fnut.2026.1860679 (PMC13457121; doi:10.3389/fnut.2026.1860679)
Supplement: Supplementary file 1 [file Table_1.docx]

**Supplementary material**

**Trends and disparities in neoplasm-related mortality with death-certificate-documented protein-energy malnutrition in the United States, 1999–2024: a CDC WONDER multiple cause of death study**

Qi Gan^1^, Kaide Xia^2^, Xiuju Yang^2*^

^1^ Department of Anesthesiology, Guizhou Aerospace Hospital, Zunyi, China.

^2^ Central Laboratory, Guiyang Maternal and Child Health Care Hospital, Guiyang Children's Hospital, Guiyang, China.

***CORRESPONDENCE**

Central Laboratory, Guiyang Maternal and Child Health Care Hospital, Guiyang Children's Hospital, Guiyang, China. Email: xjyang06@126.com

**Running title:** Neoplasm mortality and PEM trends

**Supplementary tables:**

**Supplementary Table S1. Annual proportion of neoplasm-related deaths with death-certificate-documented protein-energy malnutrition in the United States, 1999–2024, and Joinpoint trend estimates**

| **Panel A. Annual proportion of neoplasm-related deaths with documented protein-energy malnutrition** | | | |
| --- | --- | --- | --- |
| **Year** | **PEM-documented deaths, n** | **All neoplasm-related deaths, n** | **Proportion, % (95% CI)** |
| 1999 | 5,024 | 561,336 | 0.90 (0.87–0.92) |
| 2000 | 4,571 | 564,887 | 0.81 (0.79–0.83) |
| 2001 | 4,443 | 565,728 | 0.79 (0.76–0.81) |
| 2002 | 4,269 | 568,797 | 0.75 (0.73–0.77) |
| 2003 | 4,107 | 568,718 | 0.72 (0.70–0.74) |
| 2004 | 3,913 | 565,758 | 0.69 (0.67–0.71) |
| 2005 | 3,851 | 571,374 | 0.67 (0.65–0.70) |
| 2006 | 3,798 | 572,441 | 0.66 (0.64–0.68) |
| 2007 | 3,640 | 575,474 | 0.63 (0.61–0.65) |
| 2008 | 3,885 | 578,381 | 0.67 (0.65–0.69) |
| 2009 | 3,829 | 580,699 | 0.66 (0.64–0.68) |
| 2010 | 4,310 | 588,142 | 0.73 (0.71–0.75) |
| 2011 | 4,438 | 590,230 | 0.75 (0.73–0.77) |
| 2012 | 4,405 | 596,100 | 0.74 (0.72–0.76) |
| 2013 | 4,610 | 598,653 | 0.77 (0.75–0.79) |
| 2014 | 5,122 | 606,338 | 0.84 (0.82–0.87) |
| 2015 | 5,837 | 610,771 | 0.96 (0.93–0.98) |
| 2016 | 6,671 | 612,340 | 1.09 (1.06–1.12) |
| 2017 | 8,176 | 613,539 | 1.33 (1.30–1.36) |
| 2018 | 9,456 | 613,556 | 1.54 (1.51–1.57) |
| 2019 | 10,462 | 613,907 | 1.70 (1.67–1.74) |
| 2020 | 11,582 | 617,294 | 1.88 (1.84–1.91) |
| 2021 | 12,722 | 620,196 | 2.05 (2.02–2.09) |
| 2022 | 14,699 | 623,139 | 2.36 (2.32–2.40) |
| 2023 | 16,883 | 628,210 | 2.69 (2.65–2.73) |
| 2024 | 18,410 | 634,842 | 2.90 (2.86–2.94) |
|  |  |  |  |
| **Panel B. Joinpoint trend estimates for the annual proportion of neoplasm-related deaths with documented protein-energy malnutrition** | | | |
| **Period** | **APC or AAPC, %** | **95% CI** | **P value** |
| 1999–2007 | −3.76 | −6.93 to −2.27 | <0.001 |
| 2007–2013 | 3.81 | 0.13 to 8.79 | 0.047 |
| 2013–2024 | 12.92 | 12.24 to 13.95 | <0.001 |
| 1999–2024, overall AAPC | 5.14 | 4.86 to 5.50 | <0.001 |

Abbreviations: AAPC, average annual percent change; APC, annual percent change; CI, confidence interval; PEM, protein-energy malnutrition.

The numerator was the annual number of neoplasm-related deaths with protein-energy malnutrition documented in any multiple-cause-of-death field (ICD-10 E40–E46). The denominator was the annual number of all neoplasm-related deaths. Annual proportions are unadjusted percentages and should be interpreted as complementary to, rather than replacements for, age-adjusted mortality rates. The 95% CIs for annual proportions were calculated using the Wilson method. APC and AAPC were estimated using Joinpoint regression.

**Supplementary Table S2. Sensitivity analyses using alternative definitions of neoplasm-related deaths with documented protein-energy malnutrition, United States, 1999–2024**

| **Panel A. Overall temporal trends under alternative outcome definitions** | | | |  |  |  |
| --- | --- | --- | --- | --- | --- | --- |
| **Sensitivity analysis** | **Definition** | **Deaths in 1999, n** | **AAMR in 1999, per 100,000 (95% CI)** | **Deaths in 2024, n** | **AAMR in 2024, per 100,000 (95% CI)** | **Overall AAPC, % (95% CI)** |
| Sensitivity A | Malignant neoplasms only (UCD C00–C97) with documented PEM (MCD E40–E46) | 4,909 | 2.28 (2.22–2.35) | 17,915 | 5.11 (5.04–5.19) | 3.56 (3.35–3.83) |
| Sensitivity B | Malignant neoplasms only (UCD C00–C97) with documented PEM (MCD E40–E46) or cachexia (MCD R64) | 8,698 | 4.06 (3.97–4.14) | 19,756 | 5.65 (5.57–5.73) | 1.44 (1.27–1.68) |
|  |  |  |  |  |  |  |
| **Panel B. Joinpoint segment-specific trends under alternative outcome definitions** | | | | |  |  |
| **Sensitivity analysis** | **Period** | **APC, % (95% CI)** | **P value** |  |  |  |
| Sensitivity A: C00–C97 + E40–E46 | 1999–2007 | −5.09 (−6.80 to −3.96) | <0.001 |  |  |  |
|  | 2007–2013 | 1.88 (−0.80 to 5.11) | 0.13 |  |  |  |
|  | 2013–2024 | 11.34 (10.83–12.03) | <0.001 |  |  |  |
| Sensitivity B: C00–C97 + E40–E46 or R64 | 1999–2006 | −6.21 (−8.01 to −5.10) | <0.001 |  |  |  |
|  | 2006–2014 | −0.28 (−1.66 to 1.36) | 0.519 |  |  |  |
|  | 2014–2024 | 8.64 (8.11–9.38) | <0.001 |  |  |  |

Abbreviations: AAMR, age-adjusted mortality rate; AAPC, average annual percent change; APC, annual percent change; CI, confidence interval; MCD, multiple cause of death; PEM, protein-energy malnutrition; UCD, underlying cause of death.

Sensitivity A restricted the underlying cause of death to malignant neoplasms only (ICD-10 C00–C97). Sensitivity B used the same malignant-neoplasm restriction and expanded the multiple-cause definition to include documented PEM (ICD-10 E40–E46) or cachexia (ICD-10 R64). AAPCs and APCs were estimated using Joinpoint regression.

**Supplementary Table S3. Temporal robustness analyses and overlap-period comparison across CDC WONDER mortality data systems for neoplasm-related deaths with death-certificate-documented protein-energy malnutrition, United States, 1999–2024**

| **Panel A. Overall temporal robustness analyses** | | |  |  |  |  |
| --- | --- | --- | --- | --- | --- | --- |
| **Analysis** | **Analytic period** | **Maximum joinpoints** | **Overall AAPC, % (95% CI)** | **P value** |  |  |
| Primary combined-source model | 1999–2024 | 2 | 3.54 (3.28 to 3.90) | <0.001 |  |  |
| Flexible joinpoint sensitivity | 1999–2024 | 3 | 3.51 (3.29 to 3.81) | <0.001 |  |  |
| Pre-pandemic sensitivity | 1999–2019 | 2 | 1.96 (1.53 to 2.45) | <0.001 |  |  |
| Bridged-race-only sensitivity | 1999–2020 | 2 | 2.22 (1.88 to 2.63) | <0.001 |  |  |
|  |  |  |  |  |  |  |
| **Panel B. Segment-specific annual percent changes in temporal robustness analyses** | | | | | |  |
| **Analysis** | **Period** | **APC, % (95% CI)** | **P value** |  |  |  |
| Primary combined-source model | 1999–2006 | −5.58 (−9.84 to −3.88) | <0.001 |  |  |  |
|  | 2006–2013 | 1.17 (−1.29 to 5.63) | 0.214 |  |  |  |
|  | 2013–2024 | 11.43 (10.80 to 12.32) | <0.001 |  |  |  |
| Flexible joinpoint sensitivity | 1999–2007 | −5.07 (−7.32 to −3.64) | 0.018 |  |  |  |
|  | 2007–2014 | 2.20 (−3.53 to 4.43) | 0.144 |  |  |  |
|  | 2014–2018 | 14.20 (2.21 to 17.90) | 0.012 |  |  |  |
|  | 2018–2024 | 10.44 (8.69 to 11.61) | <0.001 |  |  |  |
| Pre-pandemic sensitivity | 1999–2007 | −5.09 (−8.67 to −3.56) | <0.001 |  |  |  |
|  | 2007–2014 | 2.30 (−1.08 to 6.79) | 0.124 |  |  |  |
|  | 2014–2019 | 13.82 (11.31 to 20.51) | <0.001 |  |  |  |
| Bridged-race-only sensitivity | 1999–2006 | −5.50 (−9.35 to −3.93) | <0.001 |  |  |  |
|  | 2006–2013 | 0.88 (−1.61 to 5.31) | 0.311 |  |  |  |
|  | 2013–2020 | 12.03 (10.74 to 14.36) | <0.001 |  |  |  |
|  |  |  |  |  |  |  |
| **Panel C. Comparison of bridged-race and single-race estimates during the 2018–2020 overlap period** | | | | | | |
| **Population group** | **Year** | **Bridged-race AAMR, per 100,000 (95% CI)** | **Single-race AAMR, per 100,000 (95% CI)** | **Absolute AAMR difference** | **Relative difference, %** | **95% CIs overlapped** |
| Overall | 2018 | 2.97 (2.91 to 3.04) | 2.97 (2.91 to 3.04) | 0 | 0 | Yes |
| Overall | 2019 | 3.23 (3.17 to 3.30) | 3.23 (3.17 to 3.30) | 0 | 0 | Yes |
| Overall | 2020 | 3.49 (3.43 to 3.56) | 3.49 (3.43 to 3.56) | 0 | 0 | Yes |
| White | 2018 | 2.90 (2.84 to 2.97) | 2.91 (2.85 to 2.98) | 0.01 | 0.29 | Yes |
| White | 2019 | 3.17 (3.10 to 3.24) | 3.17 (3.10 to 3.24) | 0 | 0.06 | Yes |
| White | 2020 | 3.47 (3.40 to 3.54) | 3.47 (3.40 to 3.55) | 0.01 | 0.22 | Yes |
| Black | 2018 | 4.09 (3.87 to 4.31) | 4.16 (3.93 to 4.39) | 0.07 | 1.61 | Yes |
| Black | 2019 | 4.35 (4.12 to 4.58) | 4.44 (4.21 to 4.68) | 0.09 | 2.09 | Yes |
| Black | 2020 | 4.28 (4.06 to 4.50) | 4.36 (4.13 to 4.59) | 0.08 | 1.85 | Yes |

Abbreviations: AAMR, age-adjusted mortality rate; AAPC, average annual percent change; APC, annual percent change; CI, confidence interval; MCD, multiple cause of death; PEM, protein-energy malnutrition; UCD, underlying cause of death.

The primary outcome was defined as deaths among persons aged ≥15 years with neoplasms listed as the underlying cause of death (ICD-10 C00–D48) and documented protein-energy malnutrition in any multiple-cause-of-death field (ICD-10 E40–E46). The primary model combined bridged-race data for 1999–2020 with single-race data for 2021–2024. The pre-pandemic sensitivity analysis was restricted to 1999–2019. The bridged-race-only sensitivity analysis was restricted to 1999–2020. Overlap-period comparisons were limited to Overall, White, and Black populations because the Other racial category was heterogeneous and not directly comparable between bridged-race and single-race data systems.

**Supplementary Table S4. Hispanic-origin and detailed age-specific trends in neoplasm-related mortality with death-certificate-documented protein-energy malnutrition in the United States, 1999–2024**

| **Panel A. Trends by Hispanic origin** | | | |  |  |  |
| --- | --- | --- | --- | --- | --- | --- |
| **Hispanic-origin group** | **Deaths in 1999, n** | **AAMR in 1999, per 100,000 (95% CI)** | **Deaths in 2024, n** | **AAMR in 2024, per 100,000 (95% CI)** | **Overall AAPC, % (95% CI)** | **P value** |
| Hispanic | 212 | 1.97 (1.69–2.25) | 1,463 | 3.78 (3.59–3.99) | 3.04 (2.43–3.95) | <0.001 |
| Non-Hispanic | 4,795 | 2.37 (2.30–2.44) | 16,914 | 5.42 (5.34–5.50) | 3.62 (3.37–3.94) | <0.001 |
|  |  |  |  |  |  |  |
| **Panel B. Detailed age-specific trends** | | | |  |  |  |
| **Age group** | **Deaths in 1999, n** | **Age-specific mortality rate in 1999, per 100,000 (95% CI)** | **Deaths in 2024, n** | **Age-specific mortality rate in 2024, per 100,000 (95% CI)** | **Overall AAPC, % (95% CI)** | **P value** |
| 15–44 years | 110 | 0.09 (0.07–0.11) | 401 | 0.29 (0.27–0.32) | 4.94 (4.19–5.87) | <0.001 |
| 45–64 years | 946 | 1.57 (1.47–1.67) | 3,745 | 4.54 (4.40–4.69) | 4.93 (4.41–5.66) | <0.001 |
| 65–74 years | 1,132 | 6.15 (5.79–6.51) | 5,424 | 15.30 (14.90–15.72) | 3.82 (3.48–4.21) | <0.001 |
| 75–84 years | 1,742 | 14.25 (13.59–14.93) | 5,408 | 28.02 (27.28–28.78) | 3.10 (2.91–3.36) | <0.001 |
| ≥85 years | 1,088 | 26.19 (24.66–27.80) | 3,432 | 53.33 (51.56–55.15) | 3.05 (2.75–3.41) | <0.001 |
|  |  |  |  |  |  |  |
| **Panel C. Joinpoint segment-specific annual percent changes** | | | | | |  |
| **Analysis** | **Group** | **Period** | **APC, % (95% CI)** | **P value** |  |  |
| Hispanic-origin analysis | Hispanic | 1999–2009 | −5.15 (−9.49 to −2.37) | <0.001 |  |  |
|  | Hispanic | 2009–2024 | 8.89 (7.94–10.26) | <0.001 |  |  |
|  | Non-Hispanic | 1999–2006 | −5.60 (−8.97 to −4.02) | <0.001 |  |  |
|  | Non-Hispanic | 2006–2013 | 1.18 (−1.17 to 4.85) | 0.193 |  |  |
|  | Non-Hispanic | 2013–2024 | 11.62 (11.05–12.46) | <0.001 |  |  |
| Detailed age analysis | 15–44 years | 1999–2010 | −3.26 (−6.94 to −0.43) | 0.024 |  |  |
|  | 15–44 years | 2010–2024 | 11.87 (10.34–14.08) | <0.001 |  |  |
|  | 45–64 years | 1999–2009 | −2.15 (−5.43 to 0.08) | 0.06 |  |  |
|  | 45–64 years | 2009–2024 | 9.93 (9.03–11.14) | <0.001 |  |  |
|  | 65–74 years | 1999–2007 | −4.27 (−8.47 to −2.57) | 0.003 |  |  |
|  | 65–74 years | 2007–2013 | 1.91 (−1.32 to 8.80) | 0.212 |  |  |
|  | 65–74 years | 2013–2024 | 11.27 (10.58–12.39) | <0.001 |  |  |
|  | 75–84 years | 1999–2007 | −5.69 (−7.10 to −4.61) | <0.001 |  |  |
|  | 75–84 years | 2007–2014 | 2.23 (0.10–4.48) | 0.043 |  |  |
|  | 75–84 years | 2014–2024 | 11.38 (10.81–12.16) | <0.001 |  |  |
|  | ≥85 years | 1999–2006 | −7.82 (−11.87 to −6.04) | <0.001 |  |  |
|  | ≥85 years | 2006–2013 | −1.37 (−3.98 to 3.90) | 0.316 |  |  |
|  | ≥85 years | 2013–2024 | 13.76 (13.01–14.73) | <0.001 |  |  |

Abbreviations: AAMR, age-adjusted mortality rate; AAPC, average annual percent change; APC, annual percent change; CI, confidence interval; PEM, protein-energy malnutrition.

The primary outcome was deaths among persons aged ≥15 years with neoplasms listed as the underlying cause of death (ICD-10 C00–D48) and documented PEM in any multiple-cause-of-death field (ICD-10 E40–E46). Hispanic-origin analyses were conducted independently of race classification. The age-specific mortality rates were calculated as deaths within each age group divided by the corresponding age-group population and should not be interpreted as AAMRs. Counts across Hispanic-origin groups and detailed age groups may not sum exactly to the national total because records with Hispanic origin or age not stated were not assigned to the corresponding subgroup.

| **Supplementary Table S5. State-level trends in neoplasm-related mortality with death-certificate-documented protein-energy malnutrition, 1999–2024** | | | | | | |
| --- | --- | --- | --- | --- | --- | --- |
| **State** | **1999** | |  | **2024** | | **AAPC (95% CI)** |
|  | **Counts** | **AAMR (95% CI)** |  | **Counts** | **AAMR (95% CI)** |  |
| Alabama | 83 | 2.36 (1.88, 2.92) |  | 249 | 4.67 (4.09, 5.31) | 3.38 (2.69 to 4.38)* |
| Alaska | 12 | NA |  | 34 | 5.22 (3.57, 7.52) | 2.48 (-3.78 to 9.92) |
| Arizona | 61 | 1.58 (1.21, 2.04) |  | 346 | 4.10 (3.67, 4.57) | 3.81 (2.37 to 5.35)* |
| Arkansas | 56 | 2.50 (1.89, 3.25) |  | 234 | 7.38 (6.45, 8.43) | 3.78 (2.66 to 4.99)* |
| California | 550 | 2.46 (2.26, 2.67) |  | 1540 | 4.05 (3.85, 4.26) | 2.48 (1.99 to 3.05)* |
| Colorado | 48 | 1.86 (1.37, 2.47) |  | 517 | 9.50 (8.68, 10.37) | 6.7 (5.75 to 8.37)* |
| Connecticut | 37 | 1.25 (0.88, 1.73) |  | 91 | 2.14 (1.71, 2.66) | 2.6 (1.73 to 3.62)* |
| Delaware | 21 | 3.54 (2.19, 5.42) |  | 28 | 2.15 (1.42, 3.25) | -2.27 (-3.17 to -1.37)* |
| District Of Columbia | 19 | NA |  | 22 | 3.90 (2.44, 6.00) | 8.7 (-8.75 to 31.91) |
| Florida | 249 | 1.56 (1.37, 1.76) |  | 1403 | 4.87 (4.62, 5.14) | 5.23 (4.73 to 5.89)* |
| Georgia | 135 | 2.70 (2.24, 3.16) |  | 634 | 6.11 (5.63, 6.62) | 2.88 (2.03 to 4.02)* |
| Hawaii | 25 | 2.64 (1.71, 3.90) |  | 32 | 2.00 (1.34, 2.91) | -0.48 (-2.3 to 0.78) |
| Idaho | 25 | 2.77 (1.79, 4.08) |  | 168 | 8.56 (7.29, 10.01) | 5.86 (4.3 to 8.34)* |
| Illinois | 267 | 2.86 (2.51, 3.20) |  | 456 | 3.48 (3.17, 3.83) | 1.75 (1.05 to 2.57)* |
| Indiana | 119 | 2.55 (2.09, 3.01) |  | 339 | 4.86 (4.35, 5.43) | 2.49 (1.84 to 3.21)* |
| Iowa | 48 | 1.75 (1.29, 2.33) |  | 274 | 7.88 (6.95, 8.92) | 5.52 (4.75 to 6.55)* |
| Kansas | 101 | 4.52 (3.63, 5.40) |  | 147 | 4.98 (4.20, 5.89) | 0.72 (0.01 to 1.59)* |
| Kentucky | 73 | 2.38 (1.86, 2.99) |  | 351 | 7.44 (6.67, 8.29) | 5.12 (4.52 to 5.85)* |
| Louisiana | 65 | 2.05 (1.58, 2.61) |  | 160 | 3.48 (2.96, 4.09) | 2.13 (1.07 to 3.44)* |
| Maine | 35 | 3.12 (2.17, 4.34) |  | NA | NA | -1.53 (-2.92 to 0.08) |
| Maryland | 71 | 1.88 (1.47, 2.37) |  | 274 | 4.24 (3.75, 4.79) | 1.65 (0.67 to 2.77)* |
| Massachusetts | 60 | 1.12 (0.86, 1.45) |  | 235 | 3.06 (2.67, 3.49) | 4.76 (4.1 to 5.65)* |
| Michigan | 209 | 2.81 (2.43, 3.19) |  | 509 | 4.54 (4.15, 4.97) | 1.66 (0.98 to 2.52)* |
| Minnesota | 41 | 1.06 (0.76, 1.44) |  | 462 | 7.74 (7.04, 8.50) | 7.94 (7.47 to 8.55)* |
| Mississippi | 52 | 2.49 (1.86, 3.26) |  | 101 | 3.38 (2.74, 4.14) | 2.29 (1.27 to 3.5)* |
| Missouri | 146 | 3.13 (2.62, 3.63) |  | 524 | 7.88 (7.21, 8.61) | 4.02 (3.3 to 4.81)* |
| Montana | 19 | NA |  | 83 | 6.37 (5.04, 8.02) | 3.52 (2.11 to 5.51)* |
| Nebraska | 29 | 1.97 (1.32, 2.82) |  | 200 | 10.30 (8.89, 11.89) | 6.63 (5.67 to 7.96)* |
| Nevada | 29 | 2.34 (1.55, 3.38) |  | 208 | 6.46 (5.60, 7.43) | 5.55 (3.49 to 9.24)* |
| New Hampshire | 17 | NA |  | 70 | 4.19 (3.25, 5.39) | 2.99 (1.96 to 4.63)* |
| New Jersey | 165 | 2.40 (2.03, 2.77) |  | 292 | 2.94 (2.61, 3.31) | 0.94 (0.15 to 1.86)* |
| New Mexico | 30 | 2.33 (1.57, 3.33) |  | 99 | 4.25 (3.44, 5.23) | 1.93 (0.64 to 3.4)* |
| New York | 160 | 1.06 (0.90, 1.23) |  | 377 | 1.73 (1.56, 1.92) | 2.35 (1.49 to 3.22)* |
| North Carolina | 135 | 2.32 (1.93, 2.71) |  | 681 | 6.05 (5.60, 6.53) | 4.17 (3.52 to 4.98)* |
| North Dakota | 23 | 4.01 (2.54, 6.01) |  | 66 | 8.61 (6.61, 11.12) | 3.10 (2.00 to 4.48)* |
| Ohio | 322 | 3.51 (3.13, 3.90) |  | 742 | 5.79 (5.38, 6.24) | 2.46 (1.68 to 3.12)* |
| Oklahoma | 88 | 3.16 (2.53, 3.89) |  | 214 | 5.41 (4.70, 6.21) | 2.72 (1.77 to 3.75)* |
| Oregon | 35 | 1.29 (0.90, 1.79) |  | 556 | 11.82 (10.84, 12.88) | 9.43 (8.36 to 11.27)* |
| Pennsylvania | 272 | 2.34 (2.06, 2.62) |  | 893 | 5.91 (5.52, 6.32) | 3.85 (3.41 to 4.41)* |
| Rhode Island | 20 | 2.06 (1.26, 3.18) |  | 53 | 4.22 (3.15, 5.62) | 1.99 (0.5 to 3.57)* |
| South Carolina | 93 | 3.16 (2.55, 3.87) |  | 431 | 7.23 (6.55, 7.98) | 3.34 (2.61 to 4.21)* |
| South Dakota | 16 | NA |  | 175 | 18.78 (16.03, 21.94) | 23.27 (16.91 to 40.1)* |
| Tennessee | 190 | 4.41 (3.78, 5.04) |  | 413 | 5.55 (5.01, 6.13) | 2.03 (1.1 to 3.19)* |
| Texas | 401 | 3.15 (2.84, 3.46) |  | 1872 | 7.25 (6.92, 7.59) | 3.55 (2.85 to 4.36)* |
| Utah | 32 | 2.68 (1.83, 3.78) |  | 275 | 11.32 (10.01, 12.78) | 6.33 (5.39 to 7.64)* |
| Vermont | NA | NA |  | 52 | 6.33 (4.68, 8.55) | 12.79 (1.91 to 28.05)* |
| Virginia | 137 | 2.80 (2.33, 3.27) |  | 309 | 3.36 (2.99, 3.77) | 0.88 (0.25 to 1.52)* |
| Washington | 75 | 1.80 (1.42, 2.26) |  | 680 | 8.73 (8.08, 9.43) | 7.52 (6.83 to 8.5)* |
| West Virginia | 48 | 2.87 (2.11, 3.80) |  | 72 | 3.48 (2.70, 4.48) | 0.26 (-0.96 to 1.39) |
| Wisconsin | 68 | 1.55 (1.20, 1.97) |  | 421 | 6.34 (5.74, 7.00) | 5.97 (5.01 to 7.38)* |
| Wyoming | NA | NA |  | 40 | 6.06 (4.31, 8.44) | 4.99 (-2.76 to 15.06) |

Abbreviations: AAMR, age-adjusted mortality rate; AAPC, average annual percent change; CI, confidence interval.

**Supplementary Table S6. Completeness of detailed underlying-cause neoplasm-category data and descriptive decomposition of digestive neoplasm-related deaths with death-certificate-documented protein-energy malnutrition in the United States, 1999–2024**

| **Panel A. Completeness of detailed underlying-cause neoplasm-category reconstruction** | | | | | |
| --- | --- | --- | --- | --- | --- |
| **Year** | **National total deaths, n** | **Displayed detailed-category deaths, n** | **Deaths unrepresented in detailed categories, n** | **Displayed coverage, %** | **Suppressed detailed UCD cells, n** |
| 1999 | 5,024 | 4,662 | 362 | 92.8 | 143 |
| 2005 | 3,851 | 3,495 | 356 | 90.8 | 136 |
| 2010 | 4,310 | 3,972 | 338 | 92.2 | 132 |
| 2013 | 4,610 | 4,260 | 350 | 92.4 | 127 |
| 2015 | 5,837 | 5,432 | 405 | 93.1 | 152 |
| 2019 | 10,462 | 9,987 | 475 | 95.5 | 171 |
| 2020 | 11,582 | 11,074 | 508 | 95.6 | 189 |
| 2024 | 18,410 | 17,745 | 665 | 96.4 | 229 |
|  |  |  |  |  |  |
| **Panel B. Descriptive decomposition of observed digestive neoplasm-category deaths** | | | | | |
| **Digestive subsite** | **1999, n (%)** | **2013, n (%)** | **2019, n (%)** | **2024, n (%)** |  |
| Colorectal and anus | 675 (42.2) | 525 (37.0) | 1,132 (34.3) | 1,841 (32.3) |  |
| Pancreas | 304 (19.0) | 303 (21.4) | 787 (23.8) | 1,421 (24.9) |  |
| Esophagus | 226 (14.1) | 230 (16.2) | 522 (15.8) | 941 (16.5) |  |
| Stomach | 250 (15.6) | 196 (13.8) | 336 (10.2) | 520 (9.1) |  |
| Liver and intrahepatic bile ducts | 93 (5.8) | 128 (9.0) | 368 (11.1) | 678 (11.9) |  |
| Gallbladder and extrahepatic biliary tract | 29 (1.8) | 15 (1.1) | 61 (1.8) | 107 (1.9) |  |
| Other digestive sites | 21 (1.3) | 22 (1.6) | 99 (3.0) | 194 (3.4) |  |

Abbreviations: ICD-10, International Statistical Classification of Diseases and Related Health Problems, Tenth Revision; PEM, protein-energy malnutrition; UCD, underlying cause of death.

The primary outcome was defined as deaths among persons aged ≥15 years with neoplasms listed as the underlying cause of death (ICD-10 C00–D48) and documented PEM in any multiple-cause-of-death field (ICD-10 E40–E46). National total deaths were obtained from aggregate CDC WONDER queries, whereas detailed-category deaths were reconstructed from displayed detailed UCD cells. Because CDC WONDER suppresses some small detailed cells, displayed detailed-category deaths do not necessarily sum exactly to the national total. The number of deaths unrepresented in detailed categories was calculated as the difference between the national aggregate total and the sum of displayed detailed-category deaths; it should not be interpreted as an exact count within individual suppressed cells. Digestive-subsite percentages were calculated using the sum of observed displayed digestive-category deaths within the corresponding year as the denominator. Digestive-subsite findings are descriptive only and should not be interpreted as suppression-free site-specific burden estimates or formal site-specific temporal trend analyses. All retained detailed C00–D48 code-year cells were mapped to one mutually exclusive broad neoplasm category.

| **Supplementary Table S7. Segment-specific APCs across neoplasm categories, 1999–2024** | | | | |
| --- | --- | --- | --- | --- |
| **Neoplasm Categories** | **Segment** | **Segment Start** | **Segment End** | **APC (95% CI)** |
| Breast | 0 | 1999 | 2012 | -2.32 (-4.92 to -0.27)* |
| Breast | 1 | 2012 | 2024 | 13.17 (11.58 to 15.56)* |
| Digestive organs | 0 | 1999 | 2006 | -6.04 (-13.06 to -3.63)* |
| Digestive organs | 1 | 2006 | 2013 | 2.32 (-1.70 to 8.25) |
| Digestive organs | 2 | 2013 | 2024 | 11.06 (10.14 to 13.07)* |
| Female genital organs | 0 | 1999 | 2013 | -4.63 (-7.64 to -2.24)* |
| Female genital organs | 1 | 2013 | 2024 | 15.81 (13.14 to 19.74)* |
| Head and neck / oral cavity-pharynx | 0 | 1999 | 2014 | -2.61 (-12.61 to 6.18) |
| Head and neck / oral cavity-pharynx | 1 | 2014 | 2021 | 30.07 (-6.33 to 75.88) |
| Head and neck / oral cavity-pharynx | 2 | 2021 | 2024 | 0.81 (-18.37 to 18.70) |
| Lymphoid and hematopoietic tissue | 0 | 1999 | 2010 | -3.84 (-6.91 to -1.33)* |
| Lymphoid and hematopoietic tissue | 1 | 2010 | 2024 | 12.05 (10.8 to 13.80)* |
| Male genital organs | 0 | 1999 | 2006 | -7.55 (-15.78 to -4.00)* |
| Male genital organs | 1 | 2006 | 2012 | -1.38 (-5.47 to 19.5) |
| Male genital organs | 2 | 2012 | 2024 | 12.61 (10.36 to 16.63)* |
| Other neoplasms | 0 | 1999 | 2005 | -11.13 (-21.54 to -6.49)* |
| Other neoplasms | 1 | 2005 | 2013 | 3.00 (-2.63 to 10.63) |
| Other neoplasms | 2 | 2013 | 2024 | 15.25 (13.8 to 18.2)* |
| Respiratory and intrathoracic organs | 0 | 1999 | 2007 | -6.20 (-12.21 to -3.8)* |
| Respiratory and intrathoracic organs | 1 | 2007 | 2013 | 2.24 (-3.37 to 8.88) |
| Respiratory and intrathoracic organs | 2 | 2013 | 2024 | 10.59 (9.48 to 13.54)* |
| Urinary tract | 0 | 1999 | 2011 | -2.11 (-6.31 to 0.61) |
| Urinary tract | 1 | 2011 | 2024 | 12.92 (11.28 to 15.32)* |

Abbreviations: AAMR, age-adjusted mortality rate; APC, annual percent change; CI, confidence interval.

**Supplementary Figures:**

**Supplementary Figure S1. Annual proportion of neoplasm-related deaths with death-certificate-documented protein-energy malnutrition in the United States, 1999–2024.**

The annual proportion was calculated as the number of neoplasm-related deaths with documented protein-energy malnutrition (ICD-10 E40–E46 in the multiple-cause-of-death fields) divided by all neoplasm-related deaths in the same year. Shaded bands indicate 95% confidence intervals.

**Supplementary Figure S2. Temporal trends in age-adjusted mortality rates for malignant neoplasm-related deaths with death-certificate-documented protein-energy malnutrition, with and without inclusion of cachexia, United States, 1999–2024.**

Annual age-adjusted mortality rates (AAMRs) per 100,000 population are shown for decedents aged ≥15 years and were standardized to the 2000 US standard population. Sensitivity analysis A was restricted to deaths with malignant neoplasms as the underlying cause of death (ICD-10 C00–C97) and documented protein-energy malnutrition in any multiple-cause-of-death field (ICD-10 E40–E46). Sensitivity analysis B used the same malignant-neoplasm restriction and expanded the multiple-cause definition to include either protein-energy malnutrition (E40–E46) or cachexia (R64). Lines represent annual AAMRs. Corresponding joinpoint APC and AAPC estimates are presented in Supplementary Table S2.

Abbreviations: AAMR, age-adjusted mortality rate; AAPC, average annual percent change; APC, annual percent change; ICD-10, International Statistical Classification of Diseases and Related Health Problems, Tenth Revision; PEM, protein-energy malnutrition.

**Supplementary Figure S3. Temporal robustness and continuity across CDC WONDER bridged-race and single-race mortality data systems for neoplasm-related deaths with death-certificate-documented protein-energy malnutrition, United States, 1999–2024.**

(A) Annual age-adjusted mortality rates (AAMRs) for the primary outcome, using bridged-race CDC WONDER data for 1999–2020 and single-race data for 2021–2024. The vertical dashed line denotes the transition in the primary analytic series from the bridged-race to the single-race data system. (B) AAMRs and 95% confidence intervals in the 2018–2020 overlap period, calculated separately using bridged-race and single-race files for the Overall, White, and Black populations. The primary outcome was defined as deaths among persons aged ≥15 years with neoplasms listed as the underlying cause of death (ICD-10 C00–D48) and documented protein-energy malnutrition in any multiple-cause-of-death field (ICD-10 E40–E46). Error bars indicate 95% confidence intervals.

Abbreviations: AAMR, age-adjusted mortality rate; CDC, Centers for Disease Control and Prevention; ICD-10, International Statistical Classification of Diseases and Related Health Problems, Tenth Revision; PEM, protein-energy malnutrition.

**Supplementary Figure S4. Trends in neoplasm-related mortality with death-certificate-documented protein-energy malnutrition by Hispanic origin and detailed age group in the United States, 1999–2024.**

(A) Annual age-adjusted mortality rates (AAMRs) per 100,000 population among Hispanic and Non-Hispanic decedents. (B) Annual age-specific mortality rates per 100,000 population among persons aged 15–44, 45–64, 65–74, 75–84, and ≥85 years. The primary outcome was defined as deaths among persons aged ≥15 years with neoplasms listed as the underlying cause of death (ICD-10 C00–D48) and documented protein-energy malnutrition in any multiple-cause-of-death field (ICD-10 E40–E46). Shaded bands indicate 95% confidence intervals.

Abbreviations: AAMR, age-adjusted mortality rate; ICD-10, International Statistical Classification of Diseases and Related Health Problems, Tenth Revision; PEM, protein-energy malnutrition.

**Supplementary Figure S5. Completeness of detailed underlying-cause neoplasm-category data and descriptive digestive-subsite composition among neoplasm-related deaths with death-certificate-documented protein-energy malnutrition in the United States, 1999–2024.**

(A) Comparison of national aggregate death totals with the sum of displayed detailed underlying-cause neoplasm-category deaths in selected years. Each bar represents the national total number of neoplasm-related deaths with documented protein-energy malnutrition. Each bar represents the national total number of neoplasm-related deaths with documented protein-energy malnutrition. The blue component represents deaths reconstructed from displayed detailed UCD cells, whereas the red component represents deaths unrepresented in the detailed-category reconstruction because some detailed CDC WONDER cells were suppressed or unavailable for reliable reporting.

(B) Descriptive composition of observed digestive neoplasm-category deaths in 1999, 2013, 2019, and 2024. Percentages were calculated using the total number of displayed detailed digestive-category deaths in the corresponding year as the denominator.

The primary outcome was defined as deaths among persons aged ≥15 years with neoplasms listed as the underlying cause of death (ICD-10 C00–D48) and documented protein-energy malnutrition in any multiple-cause-of-death field (ICD-10 E40–E46). Digestive-subsite findings are descriptive decompositions of displayed detailed UCD counts only and should not be interpreted as suppression-free site-specific trend estimates.

Abbreviations: CDC, Centers for Disease Control and Prevention; ICD-10, International Statistical Classification of Diseases and Related Health Problems, Tenth Revision; PEM, protein-energy malnutrition; UCD, underlying cause of death.
